# Life-Threatening Ventricular Fibrillation Linked to High-Dose Tirzepatide-Induced Gastrointestinal Side Effects

**DOI:** 10.7759/cureus.85366

**Published:** 2025-06-04

**Authors:** Thirumala Keerthi Chandrika Kammaripalle, Jacob R Giordano, Humza Rana, Amulya Gade, Sirisha Reddy

**Affiliations:** 1 Internal Medicine, Cape Fear Valley Medical Center, Fayetteville, USA; 2 Cardiology, Cape Fear Valley Medical Center, Fayetteville, USA; 3 Electrophysiology, Cape Fear Valley Medical Center, Fayetteville, USA

**Keywords:** adult cardiac arrest, cardiac arrest, cardiovascular adverse events, electrolyte disturbances, gi side effects, life-threatening arrhythmia, obesity, tirzepatide, tirzepatide glp-1, weight loss and obesity

## Abstract

Tirzepatide is a long-acting agonist of glucose-dependent insulinotropic polypeptide and glucagon-like peptide-1 receptors, used for the treatment of type 2 diabetes and obesity, and recently approved for obstructive sleep apnea. It is known to cause gastrointestinal (GI) side effects; however, these are generally not severe enough to be life-threatening. We present a unique case of a 57-year-old woman with no prior cardiac history who was on a high dose of tirzepatide (15 mg weekly) for weight loss. After her dose was increased to 15 mg, she developed severe GI symptoms, including prolonged vomiting and diarrhea, leading to profound electrolyte imbalances (K⁺ 2.2, Mg²⁺ 1.1, and corrected Ca²⁺ 5.6) and ultimately resulting in ventricular fibrillation and cardiac arrest. The patient was successfully resuscitated and stabilized with electrolyte correction. A comprehensive evaluation, including left heart catheterization, ruled out obstructive coronary artery disease. Following the cardiac arrest, the patient had severely reduced left ventricular function, likely due to hypoxia and post-cardiac arrest stunning. However, her ejection fraction significantly improved within a few weeks, suggesting that tirzepatide-associated GI side effects and subsequent electrolyte disturbances were the primary precipitating factors. This case highlights a previously undocumented risk of life-threatening arrhythmias due to severe electrolyte disturbances caused by tirzepatide-induced GI side effects. Vigilant electrolyte monitoring is crucial, particularly in patients on high doses or those with additional risk factors.

## Introduction

Tirzepatide is a dual glucose-dependent insulinotropic polypeptide (GIP) and glucagon-like peptide-1 (GLP-1) receptor agonist approved by the FDA for the treatment of type 2 diabetes mellitus, obesity, and moderate-to-severe obstructive sleep apnea in people with obesity [1-4]. Through GLP-1 receptor activation, it enhances glucose-dependent insulin secretion, suppresses glucagon release, delays gastric emptying, and promotes satiety, leading to improvements in body weight and glycated hemoglobin levels [5,6]. In addition to its classical incretin role in enhancing glucose-stimulated insulin secretion, GIP receptor activation has demonstrated several beneficial extra-pancreatic effects. Preclinical and human studies suggest that GIP, particularly when co-agonized with GLP-1, contributes to greater weight loss by promoting lipolysis, enhancing thermogenesis, and improving lipid metabolism in adipose tissue [7]. GIP receptor activation also exerts favorable cardiovascular effects, including anti-inflammatory and anti-atherosclerotic actions through modulation of macrophage activity and reduction in oxidative stress [8]. Moreover, GIP has been shown to improve insulin sensitivity in peripheral tissues, particularly adipose and skeletal muscle, and may play a synergistic role in enhancing insulin action when combined with GLP-1 receptor agonists [9]. These findings highlight the broader physiological significance of GIP signaling and support the rationale for its therapeutic targeting in dual agonist agents such as tirzepatide.

In addition to its metabolic benefits, tirzepatide has demonstrated cardiovascular advantages. Recent evidence suggests it improves outcomes in heart failure with preserved ejection fraction (EF) and reduces cardiovascular mortality and heart failure-related events [10,11].

Despite its therapeutic potential, tirzepatide is associated with gastrointestinal (GI) side effects, including nausea, vomiting, and diarrhea, which can contribute to electrolyte disturbances such as hypomagnesemia and hypokalemia [12,13]. There is evidence based on some case reports and meta-analysis that GLP1 agonists, when leading to prolonged GI side effects such as vomiting and diarrhea, can lead to secondary fluid and electrolyte disturbances [14,15]. However, severe electrolyte imbalances leading to life-threatening arrhythmias have not been previously reported, even after reviewing the post-marketing surveillance reports and going through the FDA adverse event reporting system database [16].

This case describes what is believed to be the first ever formal report of ventricular arrhythmia associated with tirzepatide use secondary to its side effects.

## Case presentation

A 57-year-old African American woman with a history of hypertension, irritable bowel syndrome, and obesity (BMI 40.9) was brought to the ED after being found unresponsive in her car by family members. Her home medications included amlodipine 10 mg daily, atorvastatin, cetirizine, linaclotide, losartan 100 mg daily, metoprolol succinate 25 mg daily, and tirzepatide 15 mg weekly. She had no prior cardiac evaluation or family history of sudden cardiac death.

The patient had been on tirzepatide therapy for one year, with gradual dose escalation, but there were no major changes in her medications. Approximately two weeks before presentation, her dose was increased to 15 mg weekly. Following this adjustment, she developed persistent GI symptoms, including frequent nausea, vomiting, and diarrhea.

**Figure 1:**
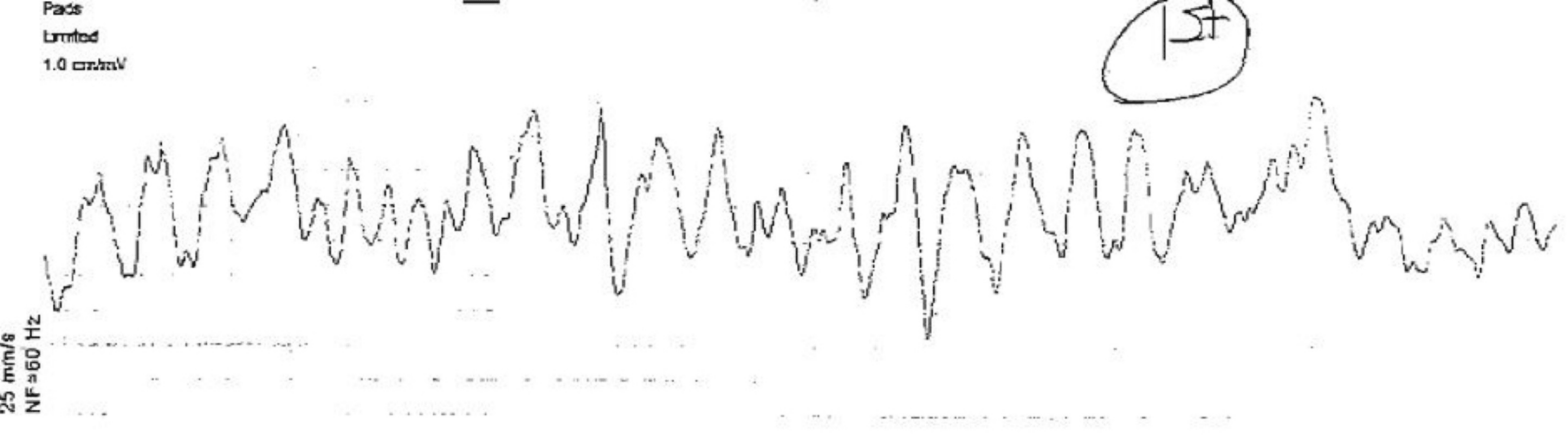
Initial rhythm strip showing ventricular fibrillation

Emergency medical services initiated CPR upon arrival after removing the patient from her car. An automated external defibrillator revealed ventricular fibrillation (VF), as shown in Figure 1.

She received two shocks and regained spontaneous circulation after two cycles of CPR. No antiarrhythmic agents were administered. She was intubated and initially stabilized at an outside hospital before being transferred to our facility for a higher level of care.

Laboratory evaluation at the outside hospital revealed severe electrolyte abnormalities, including potassium 2.2 mEq/L, magnesium 1.1 mg/dL, and corrected calcium 5.6 mg/dL. Additional laboratory values are detailed in Tables 1-4.

**Table 1:** Complete blood count

Complete blood count	Patient’s lab values	Reference ranges
White blood cell count	6.9 x 10^3^/µL	4.5-12.5 x 10^3^/µL
Red blood cell count	4.11 x 10^6^/µL	4.70-6.10 x 10^6^/µL
Hemoglobin	13.1 g/dL	13.5-18.0 g/dL
Platelet count	183 x 10^3^/µL	150-450 x10^3^/µL

**Table 2:** Comprehensive metabolic panel

Comprehensive metabolic panel	Patient’s lab values	Reference ranges
Sodium	145 mmol/L	136-145 mmol/L
Potassium	2.2 mmol/L	3.4-4.9 mmol/L
Chloride	120 mmol/L	98-107 mmol/L
Carbon dioxide	15 mmol/L	21-32 mmol/L
Blood urea nitrogen	10 mg/dL	7-25 mg/dL
Creatinine	0.50 mg/dL	0.60-1.30 mg/dL
Estimated glomerular filtration rate	>60 mL/min/1.73 m^2^	>60 mL/min/1.73 m^2^
Random glucose	187 mg/dL	74-109 mg/dL
Calcium	5.6 mg/dL	8.6-10.2 mg/dL
Aspartate transaminase	37 U/L	13-39 U/L
Alanine transaminase	62 U/L	7-52 U/L
Alkaline phosphatase	66 U/L	30-105 U/L
Total bilirubin	0.3 mg/dL	0.3-1.0 mg/dL
Total protein	7.9 g/dL	6.4-8.9 g/dL
Albumin	4.3 g/dL	3.5-5.7 g/dL
Anion gap	10 mmol/L	1-11 mmol/L

**Table 3:** Thyroid studies

Thyroid studies	Patient’s lab values	Reference ranges
Thyroid-stimulating hormone	3.685 µIU/mL	0.45-5.33 µIU/mL
Free thyroxine	0.88 ng/dL	0.61-1.12 ng/dL

**Table 4:** Other pertinent lab results

Other pertinent lab tests	Patient’s lab values	Reference ranges
Lactic acid	2.9 mmol/L	0.5-2.0 mmol/L
C-reactive protein	16 mg/L	<5 mg/L
Erythrocyte sedimentation rate	94 mm/hr	0-30 mm/hr

**Figure 2:**
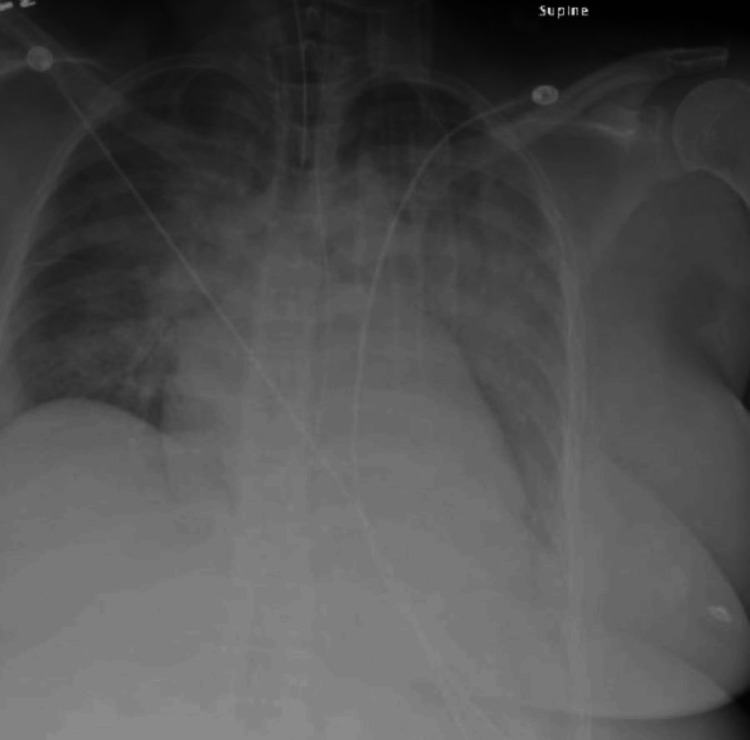
Chest radiograph showing cardiomegaly and bilateral pulmonary vascular congestion

Electrocardiography was unremarkable. High-sensitivity troponin levels were unremarkable. Urine toxicology and ethanol screens were negative. A chest X-ray revealed pulmonary edema and cardiomegaly (Figure 2), which prompted administration of IV furosemide.

**Figure 3:**
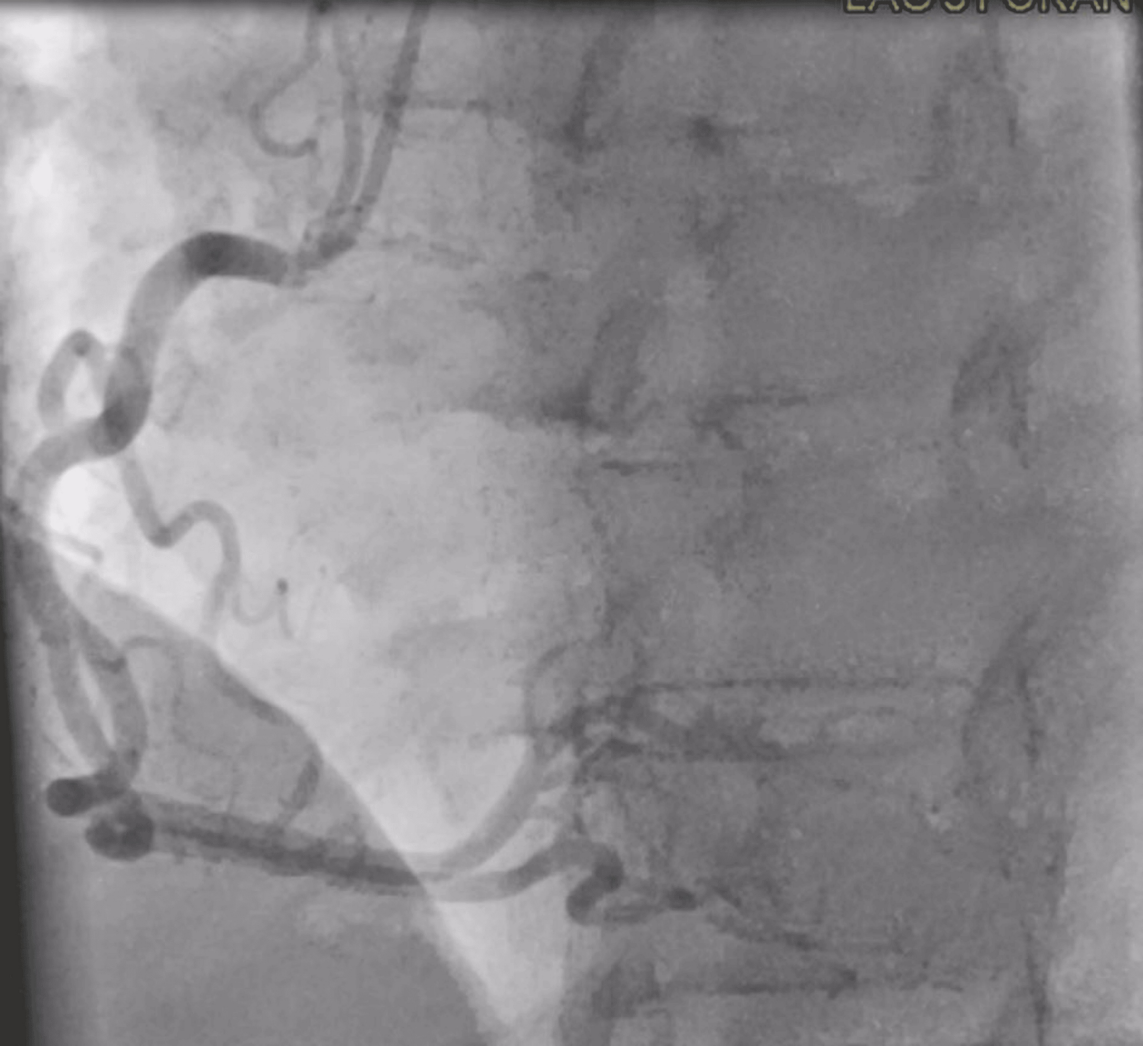
Left antero-oblique cranial view showing non-obstructive coronary artery disease in the right coronary artery

**Figure 4:**
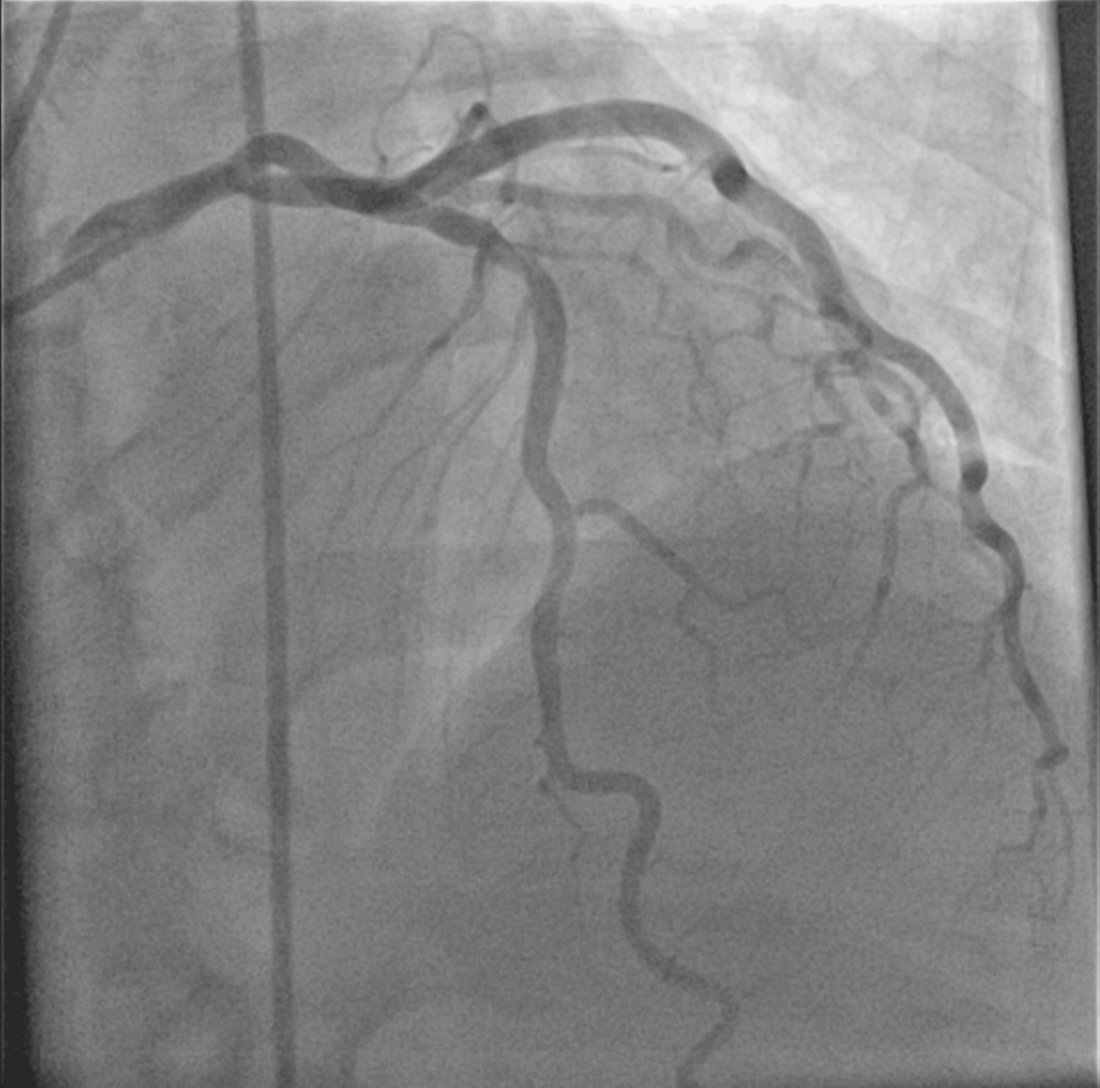
Antero-posterior cranial view showing mild, non-obstructive coronary artery disease in the left anterior descending artery

Upon admission to the ICU, the patient’s condition stabilized. Pressor support was weaned, and she was extubated without complications. Cardiac catheterization revealed mild, non-obstructive coronary artery disease, as seen in Figures 3, 4.

Transthoracic echocardiography demonstrated a reduced EF of 25%-30%, a moderately to severely dilated left ventricle, mild mitral regurgitation, mild left atrial enlargement, impaired diastolic relaxation, and no evidence of pericardial effusion or tamponade.

Electrolyte abnormalities were corrected, as shown in Table 5, and the patient was initiated on guideline-directed medical therapy for heart failure. This included empagliflozin, carvedilol, losartan, and sacubitril/valsartan (Entresto), as tolerated by her blood pressure. 

**Table 5:** Trend of electrolytes

Electrolytes	On presentation	After supplementation	At the time of discharge	Reference ranges
Potassium	2.2 mmol/L	3.4 mmol/L	4.5 mmol/L	3.4-4.9 mmol/L
Magnesium	1.1 mg/dL	2.2 mg/dL	2.3 mg/dL	1.9-2.7 mg/dL
Calcium	5.6 mg/dL	9.3 mg/dL	10.1 mg/dL	8.6-10.2 mg/dL

The patient’s hospital course was otherwise uneventful. She was discharged in stable condition, with instructions to follow up with her cardiologist and primary care provider. Her tirzepatide dose was reduced, and at outpatient follow-up, she denied having GI symptoms. A follow-up echocardiogram was performed four weeks later, which revealed improved EF.

## Discussion

VF is a common arrhythmia associated with cardiac arrest, most frequently linked to coronary artery disease. However, several other conditions can also precipitate VF, including severe electrolyte imbalances (hypokalemia, hyperkalemia, and hypomagnesemia), acidosis, hypothermia, hypoxia, cardiomyopathies, congenital long QT syndrome, Brugada syndrome, a family history of sudden cardiac death, and excessive alcohol use.

In this case, the patient had no prior cardiac history, no family history of sudden cardiac death, and no preceding cardiac symptoms such as chest pain or dyspnea. A comprehensive evaluation, including left heart catheterization, ruled out obstructive coronary artery disease or acute myocardial infarction. The primary abnormality identified was profound electrolyte disturbances, which were likely secondary to severe GI losses induced by tirzepatide. No other causes of GI losses were found.

The patient experienced persistent vomiting and diarrhea for two weeks following an increase in tirzepatide dosage from 10 mg to 15 mg weekly. According to the recommended dosing schedule, tirzepatide is initiated at 2.5 mg weekly and increased in 2.5 mg increments every four weeks until the maximum tolerated dose is reached, with 15 mg being the highest approved dose [17]. A systematic review by Mishra et al. found that GI adverse effects were most prevalent at the 15 mg dose, with a significant dose-dependent relationship between tirzepatide dose and the incidence of GI symptoms [18]. While the patient had previously tolerated 5 mg and 10 mg doses without significant adverse effects, the escalation to 15 mg was associated with severe nausea, vomiting, diarrhea, and loss of appetite, leading to dehydration and critical electrolyte imbalances. Infectious and other alternative causes of her symptoms were ruled out. Even though the patient’s home medication list included linaclotide, she has used it as needed for many years, and she has not taken it in recent times.

The patient presented with profound hypokalemia and hypomagnesemia, well-recognized risk factors for ventricular arrhythmias, including VF [19,20]. Her left heart catheterization showed non-obstructive coronary artery disease. Following cardiac arrest, she exhibited severely reduced left ventricular function with global hypokinesis, most likely due to post-cardiac arrest myocardial stunning and hypoxia. However, her EF improved significantly within weeks, further supporting the hypothesis that severe electrolyte disturbances were the primary trigger for her arrhythmia.

Although direct evidence linking tirzepatide to life-threatening arrhythmias is lacking, this case illustrates a plausible mechanism by which its GI side effects can lead to critical electrolyte depletion and subsequent cardiac events. It underscores the need for heightened vigilance when prescribing tirzepatide, particularly at higher doses or in patients with additional risk factors such as irritable bowel syndrome. Proactive electrolyte monitoring and ECG surveillance should be considered for patients experiencing persistent GI symptoms to mitigate the risk of fatal arrhythmias.

## Conclusions

This case highlights the rare but serious risk of severe electrolyte imbalances associated with the GI side effects of tirzepatide. The profound hypokalemia, hypomagnesemia, and hypocalcemia observed in this patient directly contributed to VF and cardiac arrest.

A comprehensive evaluation excluded alternative etiologies, strongly implicating tirzepatide-induced electrolyte disturbances as the primary precipitating factor. While tirzepatide remains a highly effective therapeutic option for glycemic control and weight loss, it underscores the importance of close monitoring of electrolyte levels in patients receiving tirzepatide or GLP1 agonists, particularly at higher doses or in those with additional risk factors, such as irritable bowel syndrome or chronic diuretic use. Early recognition of severe GI side effects and proactive management of electrolyte imbalances are critical to preventing serious complications. Further research is warranted to explore the relationship between tirzepatide, its side effects, and the risk of fatal arrhythmias, ensuring safer use of this increasingly popular medication.
